# High-Columbic-Efficiency Lithium Battery Based on Silicon Particle Materials

**DOI:** 10.1186/s11671-015-1103-0

**Published:** 2015-10-08

**Authors:** Junying Zhang, Chunqian Zhang, Shouming Wu, Xu Zhang, Chuanbo Li, Chunlai Xue, Buwen Cheng

**Affiliations:** State Key Laboratory on Integrated Optoelectronics, Institute of Semiconductors, Chinese Academy of Sciences, Beijing, 100083 China; Zhejiang Fluoride and Silicon Research Institute, Quzhou, Zhejiang 324100 China

**Keywords:** Polycrystalline silicon particle, High columbic efficiency, Lithium-ion battery

## Abstract

Micro-sized polycrystalline silicon particles were used as anode materials of lithium-ion battery. The columbic efficiency of the first cycle reached a relatively high value of 91.8 % after prelithiation and increased to 99 % in the second cycle. Furthermore, columbic efficiency remained above 99 % for up to 280+ cycles. The excellent performances of the batteries were the results of the use of a proper binder to protect the electrode from cracking and the application of a suitable conductive agent to provide an efficient conductive channel. The good performance was also significantly attributed to the electrolyte in the packaging process.

## Background

Silicon is considered as a promising candidate for anode materials in Li-ion battery owing to its high theoretical capacity (4200 mAh/g, ten times higher than graphite) [1], low lithium alloying/dealloying potential (about 370 mV vs. Li/Li^+^) [2], and long discharge plateau [3]. The abundant reserves also increase silicon’s advantage in commercial application. However, the low conductivity and the huge volume expansion (400 % in maximum) [4] usually cause poor cycle stability. Nanostructured Si materials are among the most effective strategies to address these issues and are attracting increasing attention [5–11]. However, high cost, low product yield, poor stability, and low first columbic efficiency (CE) because of the large superficial area limit its application. Coating of shell materials on the surface is another method to improve the cycle characteristic [12–15]. The shell can act as a protective layer that accommodates the volume extension and provides a conductive agent [16–20]. However, a match between the core and shell materials and the uniform coating are hard to realize. In this paper, we apply the polycrystalline silicon particles with sizes of about 4 μm as anode materials to fabricate the battery. The CE of the battery reaches a relatively high value of 91.8 % in the first cycle after prelithiation and increases to 99 % in the second cycle. The CE also remains above 99 % for up to 280+ cycles. The excellent performance is ascribed to the application of a suitable conductive agent and binder that provide the efficient conductive channel and protect the electrode from cracking. Furthermore, the electrolyte in the packaging process is the key to a good performance.

## Methods

The polycrystalline Si particles (AR) of about 4 μm, conductive materials [graphite, super P carbon black, vapor grown carbon fibers (VGCF) (AR)], and a polyacrylic acid (PAA) (AR, Sigma) binder with a weight ratio of 6:3:1 were thoroughly mixed with deionized water by a magnetic stirrer for 6 h. Anodes with one of the graphite, super P carbon black, and VGCF were used as conductive materials for comparison. Mixed slurry was then coated onto copper foil with a thickness of 150 μm. The slurry was ready for cell assembly after heating at 110 °C for 20 h in a vacuum drying oven.

Coin-type half cells (2025R type) with lithium foils as counter electrodes were assembled in a glove box (Mikrouna Super 1220/750) in an argon atmosphere. The electrolyte was LiPF_6_ (1 M) in ethylene carbonate/methyl ethyl carbonate [EC/EMC, 30:70 vol% (AR)] with fluoroethylene carbonate (FEC (AR)) as additive. A comparison battery without FEC as additive was also assembled. Glass fiber filter was used to stabilize the coin system. Other samples with different conductive agents or electrolytes were prepared using the same procedure.

The coin-type half cells were cycled at a rate of 0.2 C on land battery test system with one cycle prelithiation. The process has a comparatively large discharging capacity of 800 mAh/g. The cyclic voltammetry (CV) spectroscopy (in a range of 20 to 2.7 V and at a rate of 0.1 mV/s) and electrochemical impedance spectroscopy (EIS, in a range of 100,000 Hz to 0.01 Hz at a magnitude of 0.05 mV) were measured with an electrochemical workstation (PGSTAT302N, Autolab). A scanning electron microscope (SEM) was utilized to investigate the morphology of Si particles before and after cycling and the uniformity of anodes.

## Results and Discussion

**Fig. 1:**
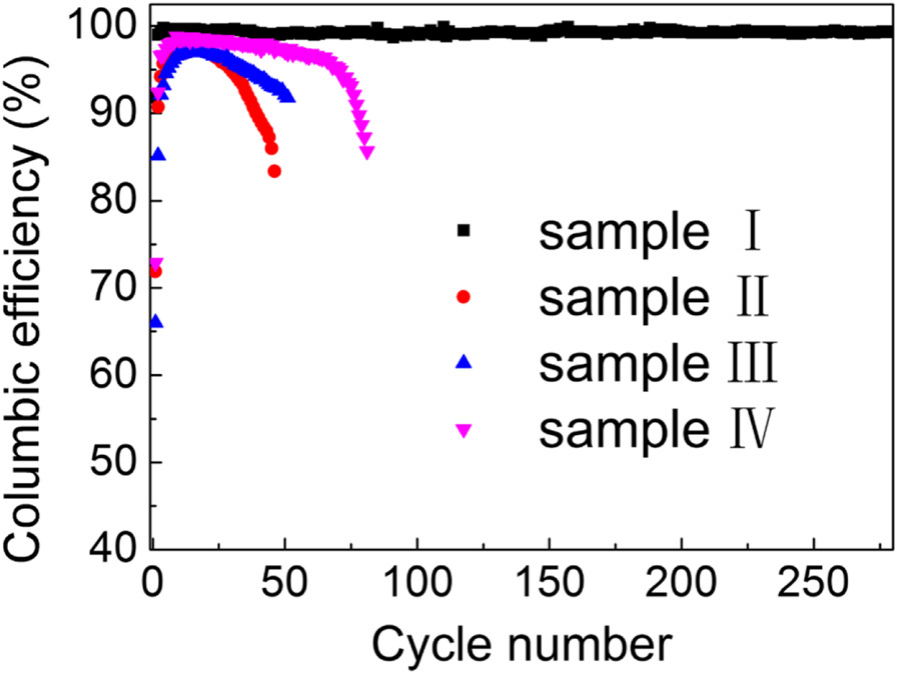
Columbic efficiency of samples with different conductive agents

**Fig. 2:**
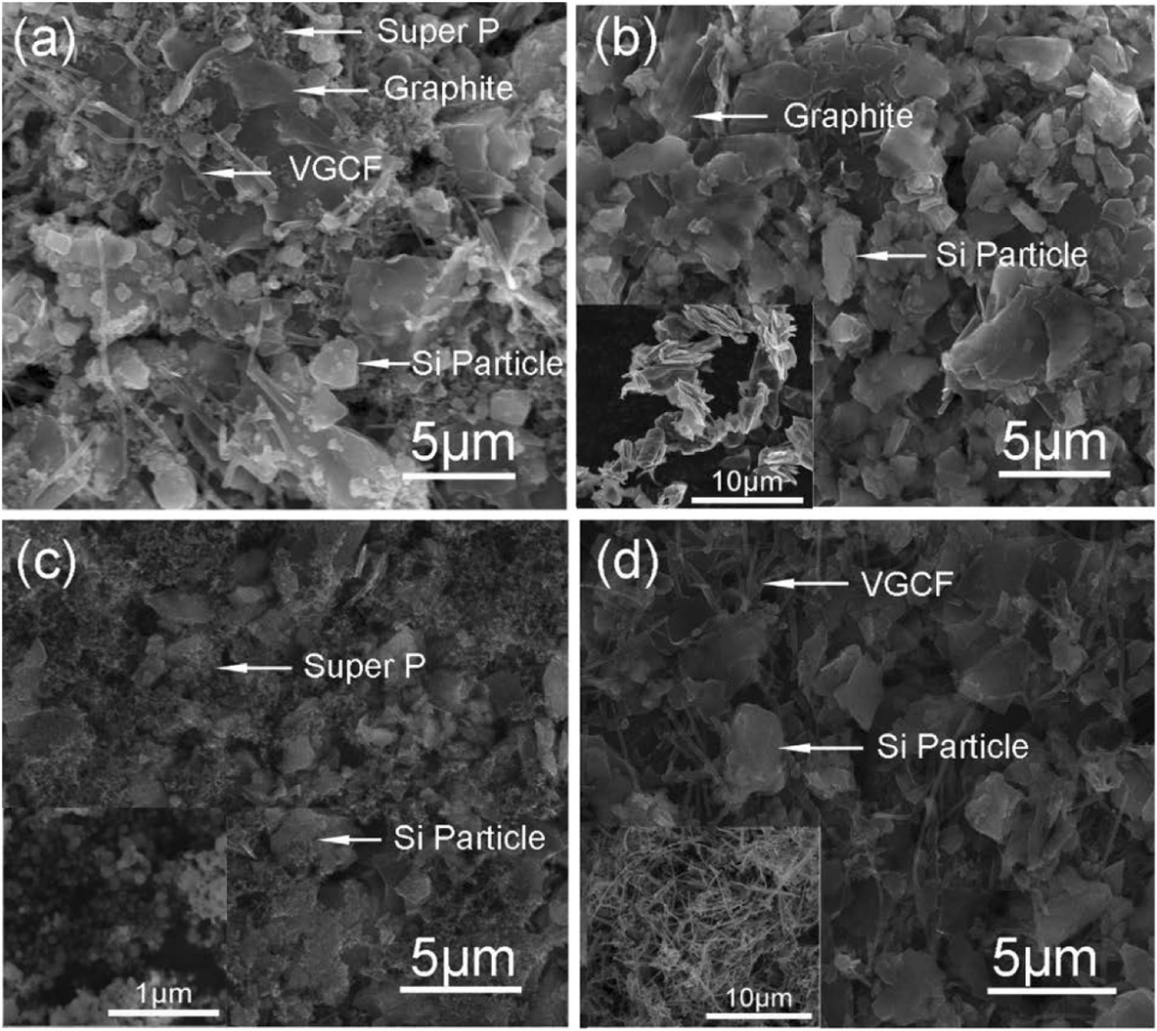
SEM of the anode material of samples I (**a**), II (**b**), III (**c**), and IV (**d**). Graphite (*inset* of **b**), Super P (*inset* of **c**), and VGCF (*inset* of **d**)

Experimental results indicate that the conductive agents play an important role in improving the performance of batteries. The battery with the conductive agents of graphite, super P carbon black, and VGCF labeled as sample I exhibits the best cycle performance. As shown in Fig. 1, the CE reaches a high value of 91.8 % in the first cycle after prelithiation, and this value increases to 99 % just in the second cycle and remains above 99 % up to 280+ cycles. For a better comparison, several batteries with different conductive agents of graphite, graphite + Super P carbon black, and graphite + VGCF labeled as samples II, III, and IV, respectively, were also prepared. The good cycle performance of sample I is believed to originate from the combination of the advantages of the three conductive agents. As shown in the SEM images of the anode materials (in Fig. 2), the VGCF forms a conductive network that guarantees the transmission of electron between particles. The application of graphite helps hinder the aggregation of Si particles for its larger dimension. The super P carbon black, which is about 100 nm small, effectively fills the space between Si and graphite particles. The combination of the three conductive agents provides good conductive network as well as protective network to avoid the aggregation of Si anode.

**Fig. 3:**
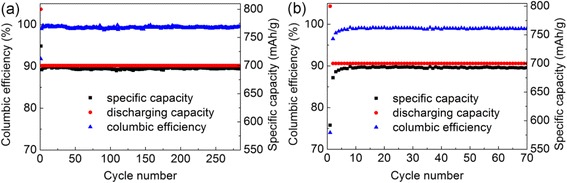
Specific capacities and columbic efficiencies of batteries with additives of FEC (30 % in weight) (**a**) and without VC and FEC additives (**b**)

**Fig. 4:**
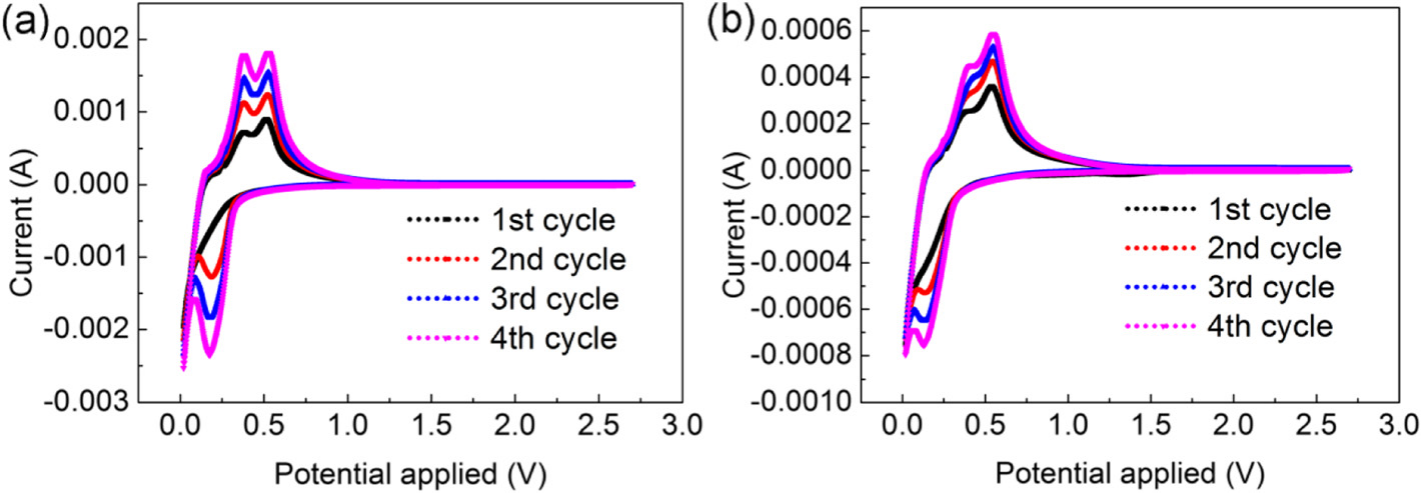
CV curves of cells with additives of FEC (**a**) and cells without FEC additive (**b**)

**Fig. 5:**
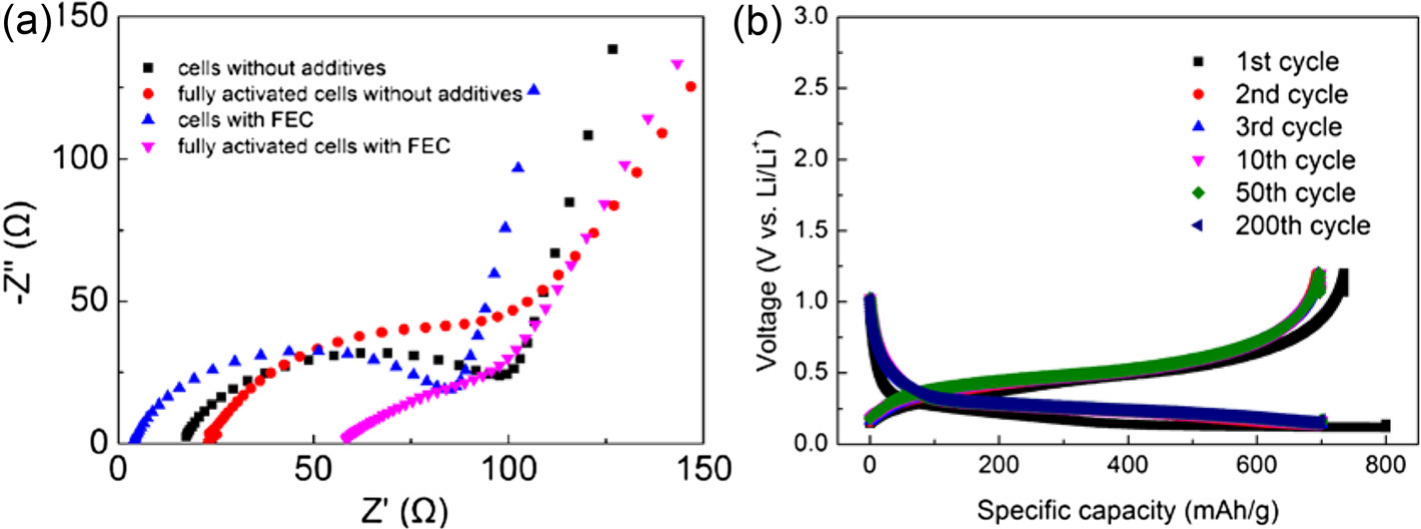
**a** Impedance spectroscopy of pristine and activated cells without FEC additives and cells with additives of FEC. **b** Voltage vs. capacity spectroscopy of cells with additives of FEC of different cycles

The experiment shows that the additives in electrolyte significantly contribute to the cycling performance of the battery. The CE of cells with additives of FEC [30 % in weight, Fig. 3a] is higher than those of cells without additives (Fig. 3b). From their CV spectroscopies in Fig. 4, a small shift is found in the redox peak potential from 0.13 V of the cells without additives to ~0.19 V of the cells with additives. This result means the electrolyte additives decompose first at a relatively higher voltage to form a stable solid electrolyte interface (SEI) on the surface of the Si anode, thereby hindering the decomposition of the electrolyte [21, 22]. The formation of SEI guarantees a lower irreversible capacity as shown in Fig. 3a. At the same time, the current value of the cells with additives of FEC is evidently larger than those of the cells without additives, corresponding to a higher battery capacity. Moreover, the integrity of materials is also in accordance with the study of impedance spectroscopy (Fig. 5a). Before discharging, the impedances of the two cells are similar as electrolyte additives had no contribution to the conductance. However, after the activation process, the impedance values significantly differ, thereby conforming with our discussion above that the comparatively intact anode with the protection of the stable SEI has much better conduction in the cycling process. Moreover, a stable and elastic SEI formed at 0.19 V in the discharging process keeps the anode material from fragmenting.

The voltage versus capacity spectroscopy of the Si anode battery with a conductive mixture (graphite/Super P carbon black/VGCF = 1:1:1) and electrolyte additives (FEC) is depicted in Fig. 5b. The initial voltage is about 1.0 V, which is relatively lower than that of the common value (about 3.0 V) because of the electrochemical prelithiation before cycling. This process helps form a stable SEI before cycling and provides an effective channel for Li ions. Consequently, the reversible capacity in the first cycle is promoted. The high CE in the first cycle is especially beneficial to whole cell with limited Li ions. The curve slightly changes from the 2nd cycle to the 200th cycle, thereby indicating the significant stability of the anode material. The long plateau assures a stable voltage output.

## Conclusions

Micro-sized polycrystalline silicon particles are used as anode material of lithium-ion battery. The CE of the first cycle reached a relatively high value of 91.8 % after prelithiation and increased to 99 % in the second cycle. Moreover, the CE remained above 99 % for up to 280+ cycles. The suitable conductive agent and binder showed significant contribution to stability and to excellent cycling performance. The conductive agent, which composes of graphite, Super P carbon black, and VGCF, provided an efficient conductive channel and kept the anode material from agglomerating. The electrolyte in the battery was also critical in obtaining a good performance, and those with FEC as additives could hinder the decomposition of electrolyte and help form a stable SEI on the surface of anodes. Thus, continuous consumption of Li ions is prevented. The prelithiation process helped form an SEI before cycling, thereby decreasing the irreversible capacity in the first cycle and led to higher CE.

## Abbreviations

CE: columbic efficiency
CV: cyclic voltammetry
EC: ethylene carbonate
EIS: electrochemical impedance spectroscopy
EMC: methyl ethyl carbonate
FEC: fluoroethylene carbonate
PAA: polyacrylic acid
SEI: solid electrolyte interface
SEM: scanning electron microscope
